# Exploring Potential Distribution and Environmental Preferences of Three Species of *Dicranomyia* (Diptera: Limoniidae: Limoniinae) Across the Western Palaearctic Realm Using Maxent

**DOI:** 10.3390/insects16101022

**Published:** 2025-10-02

**Authors:** Pasquale Ciliberti, Pavel Starkevich, Sigitas Podenas

**Affiliations:** State Scientific Research Institute Nature Research Centre, Akademijos Str. 2, LT-08412 Vilnius, Lithuania; pavel.starkevic@gamtc.lt (P.S.); sigitas.podenas@gamtc.lt (S.P.)

**Keywords:** environmental space, evaluation, species distribution models, transferability, variable importance, uncertainties

## Abstract

Species distribution models were built for three short-palped crane fly species of the genus *Dicranomyia*: *Dicranomyia affinis*, *Dicranomyia chorea*, and *Dicranomyia mitis*. The main objective of this study was to assess potential habitat suitability in undersampled regions and explore differences in environmental space. *Dicranomyia affinis* was historically considered a variety of *Dicranomyia mitis* due to their morphological similarity. In contrast, *Dicranomyia chorea* is a widespread species. The biology and ecology of these species remain poorly understood. Models were developed using Maxent, a widely used tool. Our results indicate that *Dicranomyia affinis* and *Dicranomyia chorea* share highly similar predicted habitat suitability, with high suitability across the Mediterranean, Central, and Northern Europe, moderate suitability in Eastern Europe, and low suitability in Central Asia. In contrast, *Dicranomyia mitis* is predicted to have greater habitat suitability in Eastern Europe and Scandinavia, with lower suitability in Mediterranean regions. Analysis of variable importance revealed possible ecological differences between the species. While climatic factors primarily influenced the models for *Dicranomyia affinis* and *Dicranomyia chorea*, *Dicranomyia mitis* was more strongly influenced by the variable pH. These findings may provide insights into potential distributions in undersampled areas and improve our understanding of the species’ ecology.

## Simple Summary

One of the most fundamental questions in biology relates to understanding the drivers that shape observed species distributions. Various methods based on statistics and machine learning, collectively known as Ecological Niche Models or Species Distribution Models, have been proposed to address this question. These tools aim to predict distributions by using environmental information from areas where the species is known to occur and comparing them to data from areas where the species has not yet been observed. Maxent is one of the most popular and user-friendly tools for this purpose. In this study, we explore the potential distributions of three closely related species of *Dicranomyia* crane flies, *Dicranomyia affinis*, *Dicranomyia chorea*, and *Dicranomyia mitis*, in the Western Palaearctic realm using Maxent. Our results indicate that *Dicranomyia affinis* and *Dicranomyia chorea* have distributions that are very similar to each other, but different from that of *Dicranomyia mitis.* Additionally, there appear to be differences in the environmental variables that most influence their potential distributions. The models for *Dicranomyia affinis* and *Dicranomyia chorea* are primarily influenced by two climatic variables, while the model for *Dicranomyia mitis* is mostly influenced by soil pH.

## 1. Introduction

One of the fundamental questions in ecology relates to understanding what drivers shape observed distributions of species. Understanding what causes spatial distribution is essential for predicting the impact of abiotic or biotic changes. Anthropogenic impact is exerting significant pressure on ecosystems. Human influence on nature is perceived as so great that for some authors, we are living in the Anthropocene era [1]. A useful concept to describe species requirements is the niche concept [2]. Since its introduction [3], the concept has changed from simply referring to habitat conditions to a multidimensional abstract hypervolume. Hutchinson [4] defined the niche as the conditions required by a species to survive in a multivariate environmental space. This differs from what we observe due to limitations imposed by interactions with other species. Therefore, Hutchinson [4] differentiated between the fundamental and realized niche. Estimation of the fundamental niche is an exercise of great value for conservation biology. The estimated fundamental niche can be projected into geographic space to predict distributions or changes in spatial patterns due to anthropogenic impact (i.e., climate change), biological invasions, or to identify areas suitable for conservation. However, estimation of the fundamental niche is not an easy task.

Ecological Niche Modeling (ENM) is a correlative method that attempts to estimate the fundamental niche of a species. The method relates known occurrences to environmental variables to define the environmental space of the species of interest. Subsequently, this prediction is projected in geographic space [5].

There are many different algorithms for this purpose based on statistical theory or machine learning.

The method has been used to predict current distribution in geographic space, biological invasions [6], and future distributions under climate change [7].

Spatial distribution modeling (SDM) is another term often used to describe the correlative distribution models. Users of this term appreciate that their input data is not sufficient to estimate the fundamental niche of the species studied.

It is, in fact, difficult to estimate the fundamental niche from observed occurrences only because those occurrences represent only the geographic space reached by the species. In other words, dispersal constraints limit the portion of the suitable environment occupied by the species. This is also true for biotic interactions. Dispersal and biotic interactions are seldom incorporated into models, and the set of points used to model distribution is often biased, further reducing the portion of the fundamental niche that is modeled.

In addition, some occurrence points could be sampled from sink populations, which describe habitat conditions in which the population would go extinct without immigration [8].

The same applies to environmental variables. Only a subset of the influential environmental variables is modeled. Ideally, one should try to model the most relevant variables for the species studied. This implies a thorough knowledge of the biology and ecology of the species, which for many arthropods is not there.

The two terms are often used as synonyms. This is logical because researchers using the two different definitions rely on the same tools and often publish in the same journals.

Other terms have been used as well, for example, bioclimatic envelope models [9] or habitat suitability models [10].

Some authors opt for a stricter distinction between the two most used definitions, arguing that ENM should be used for models that attempt to estimate the fundamental niche and make predictions in other geographic areas or time periods, while SDM should be used for models making geographic predictions in current time periods and geographic space.

Throughout this article, we will use the term SDM for the models that we produce because we sense that our sample points are very biased and probably not capturing the full range of conditions to describe the fundamental niche of the species studied.

*Dicranomyia* Stephens, 1829 is one of the most speciose genera of Limoniidae, with over 1000 described species [11]. However, the biology and ecology of this species-rich genus remain poorly understood.

In this study, we aim to model the habitat suitability of three *Dicranomyia* species in the Western Palaearctic realm: *Dicranomyia affinis* (Schummel, 1829), *Dicranomyia chorea* (Meigen, 1818), and *Dicranomyia mitis* (Meigen, 1830), and to test which variables contribute most to the predicted distribution. These species belong to the *Dicranomyia chorea* species group, which was established by Starý [12] based on a few morphological characters, such as the length of the cervical sclerites and antepronotum, as well as the position of the subcostal vein 2 relative to the second anal vein.

*Dicranomyia mitis* was historically considered a common and widespread species. However, Starý and Stubbs [13] elevated four previously recognized varieties of *D. mitis* to species level, also reinstating *D. affinis* in the process. *Dicranomyia affinis* and *D. mitis* are structurally very similar and are primarily distinguished by their color patterns [13]. This morphological similarity makes studying their potential distributions and ecological preferences particularly interesting. Indeed, while models are prone to uncertainties, differences in distributions or ecological preferences could give some insights into taxonomical differences between the two species.

*Dicranomyia chorea* is widely distributed, and we expect its predicted geographic range to be extensive. We also expect the distributions of *D. affinis* and *D. mitis* to be extensive, and additionally, we expect the three distributions to overlap.

To model the spatial distribution of these species, we used Maxent, one of the most widely used [14] and user-friendly SDM tools [15]. Maxent has been shown to perform well [16]; however, no single tool consistently outperforms others across all aspects of modeling [17].

Maxent predicts species distributions using known occurrence points and a set of environmental variables [15]. These variables are often climatic but other types of factors can be included as well, for example, variables describing soil types, vegetation characteristics, or variables describing biotic interactions such as competition or predation.

Since Maxent is a presence-only modeling tool, it builds distribution models by comparing presence locations with background points (randomly selected from the study area). Users can define the number of background points used in the model [18].

Maxent applies various transformations to environmental predictors, referred to as features. Five feature types are available: linear, quadratic, product, hinge, and threshold. Users can control the complexity of the models by selecting the feature transformations they want to include [18]. By default, Maxent uses the Auto Feature option, which automatically selects different transformations depending on the number of presence points. This can result in complex models [18].

The core of Maxent’s method involves estimating a probability density function from the presence points and comparing it to the probability density function estimated across the entire study area from the background points. The resulting predicted distribution is the most spread-out distribution constrained by the density function of the presence points [16].

To avoid overfitting, Maxent uses a regularization parameter. Users can adjust this parameter to control model complexity. The aim of the study is obviously important in deciding which settings to use. For example, researchers focused on identifying environmental conditions for conservation purposes might prefer models closely fitted to presence points. Conversely, modelers aiming to predict species distributions outside the known range, or interested, as in our case, in exploring potential geographic distributions may favor models that are more generalized.

## 2. Materials and Methods

### 2.1. *Occurrence* Points and Study Area

**Figure 1:**
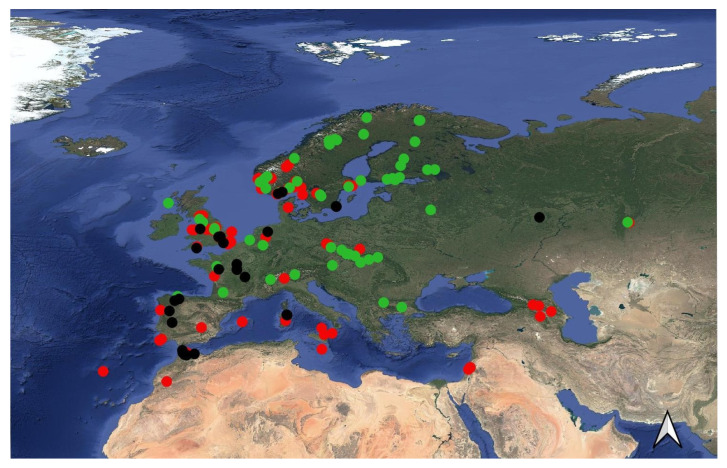
Occurrence localities of the species modeled. Black dots = *D. affinis*, Red dots *= D. chorea*, Green dots *= D. mitis.* The arrow indicates north.

Occurrence points were downloaded from the Global Biodiversity Information Facility website (GBIF) [19], filtering out records without geographic coordinates and selecting only preserved specimens. This resulted in 41 points for *D. affinis* [20], 126 points for *D. chorea* [21], and 96 points for *D. mitis* [22]. This dataset contained multiple records with identical coordinates. To avoid redundancy, we retained only one point per locality, using the R package (R software, version 4.5.2, R Core Team) [23] rgbif version 3.8.3 [24]. This dataset was supplemented with records from the literature (Table S1). Additionally, a few specimens were georeferenced from the entomological collection of the Naturalis Biodiversity Center in Leiden, The Netherlands (RMNH). Furthermore, one *D. chorea* specimen was collected during an excursion to Aspromonte National Park, Italy, in June 2024. For literature records lacking geographic coordinates, we georeferenced the data ourselves using the Point-Radius Method as recommended by Wieczorek et al. [25]. Coordinates and radius calculations were obtained via Google Maps, and only records with an estimated accuracy within a 5 km radius were included (Table S1). The final dataset comprised 30 points for *D. affinis*, 75 for *D. chorea*, and 53 for *D. mitis* (Figure 1). The study area corresponds approximately to the Western Palaearctic realm, encompassing Europe, North Africa, the Middle East, and the Caucasus. The selected geographic extent was (xmin = −28.33333, xmax = 71.04167, ymin = 19.54167, ymax = 84.33333). This region was chosen to assess potential habitat suitability in areas where faunistic and distributional data are scarce compared to Central and Northern Europe.

### 2.2. Environmental Variables

Environmental variables derived from monthly temperature and rainfall data were downloaded from WorldClim [26]. The 19 bioclimatic variables were obtained at a resolution of 2.5 arc-minutes to accommodate uncertainties in our self-georeferenced points. Additionally, soil characteristic variables were downloaded from SoilGrids [27], including clay content, nitrogen, pH, sand, silt, and soil organic carbon content at a depth of 15–30 cm. These variables were included because *Dicranomyia* larvae develop in moist or aquatic habitats, where soil characteristics may directly or indirectly influence their development. Obviously, soil characteristics also influence vegetation.

Soil variables were downloaded at a 5 km resolution and subsequently clipped to match the extent of the bioclimatic variables using QGIS (version 3.36.2; QGIS Development Team, 2009) [28]. The study area was created by clipping both climatic and soil variables using a custom polygon created in QGIS. The soil variables were reprojected to the WGS 84 coordinate system, and the cell size was adjusted from 5000 to 0.041 using the warp function with average as the resampling method. Collinearity between predictor variables can lead to unnecessarily complex models and redundant information. Although Elith et al. [29] suggest that machine learning methods are less affected by collinearity, Dormann et al. [30] caution that it can still pose problems when predictions encompass areas with a different collinearity structure. To address this, we removed variables with a correlation coefficient of >0.7. Correlation calculations were performed using the ENMTools package, version 1.5.1, in R [31]. We retained nine variables, of which five are bioclimatic and four represent soil characteristics (Table 1).

### 2.3. Modeling Approach and Evaluation

We used the stand-alone version 3.4.4 of Maxent.

Users of Maxent can either use the default settings [32] or manually adjust the model parameters. To determine the optimal settings for our study, we built models with different combinations of feature types and regularization multipliers. The feature types tested included Linear (L), Quadratic (Q), Linear Product (LP), Linear Quadratic (LQ), and Quadratic Product (QP). We evaluated five regularization multipliers: 1, 2, 3, 4, and 5. Beyond these model parameters, we also tested the effect of background point selection on predictions. Background points represent the environmental conditions potentially encountered by the species across the study area, helping to define habitats that favor suitability. We generated two sets of models: one using the default 10,000 background points (10k) and another using 500,000 background points (500k). In total, we produced 150 models, 50 for each species, by systematically combining feature types, regularization values, and background point settings.

Model evaluation is a crucial step in species distribution modeling. However, there is no consensus on the best evaluation metric to use. We used two metrics: the widely used Area under the Receiver Operating Characteristic Curve (AUC) and the Akaike Information Criterion corrected for small samples (AICc).

The concept behind AUC is straightforward: a well-performing model should correctly classify presence points as present and background points as absent. The AUC metric ranges from 0 to 1, with values above 0.5 indicating that the model predicts the species distribution better than random. We set aside 50% of the occurrence data as a test subset and applied the Maxent subsampling option to evaluate the produced models. Specifically, for *D. affinis*, we used 15 points for training and 15 for testing; for *D. chorea*, 38 points for training and 37 for testing; and for *D. mitis*, 27 points for training and 26 for testing.

Ideally, model evaluation should be conducted using independent data collected through a different approach. However, this is often not feasible, and we acknowledge that our test data share the same biases as our training data. When test data are set aside, Maxent calculates two AUC values: Training AUC and Test AUC.

We used Test AUC to evaluate our models, as it is less prone to overfitting [14].

Additionally, we applied the AICc, following Warren and Seifert [14]. AICc evaluates how well the model fits the data and helps identify the model that excludes redundant parameters.

AICc was calculated using the function aic.maxent of the R package ENMeval version 2.0.5.2. [33].

The argument askoverwritefile was not used. This was to ensure that Maxent used the same background points for all models and this way we could compare models with each other.

Another important choice that Maxent users have to make is the choice of the output format. We chose the raw format because the logistic output relies on the assumption of a species prevalence of 0.5, which is a questionable assumption [18].

The best-performing model parametrization for our study was determined by selecting the settings with the highest Test AUC and lowest AICc values. Once the optimal settings were identified, we built final models using all available occurrence points, resulting in two final models for each species.

### 2.4. Most Influential Variables

The three variables with the highest influence on the models were retained from the Maxent output. We considered the permutation importance estimate and the Jackknife test of the Maxent output. The permutation importance is calculated by randomly changing the values of the variable on training points and background points and calculating the relative drop in training AUC. Additionally, Maxent produces a Jackknife test in which variable importance is assessed using the gain. The gain is a measure of the model’s ability to correctly distinguish between presence points and background points [15]. The Jackknife test in Maxent calculates the gain when the variable is used alone and when the model is built without that variable.

A Principal Component Analysis (PCA) of the most influential variables was conducted. The scores of the principal components were visualized to inspect whether the environmental space of the most influential variables was different between the species considered. The PCA was calculated in RStudio version 4.4.2 (www.rstudio.org, accessed on 20 February 2025) [34].

### 2.5. Sampling Bias

The data used to train models should accurately represent the environmental conditions that favor species presence. However, this is rarely the case, as occurrence points used in model building are often heavily biased [35].

**Figure 2:**
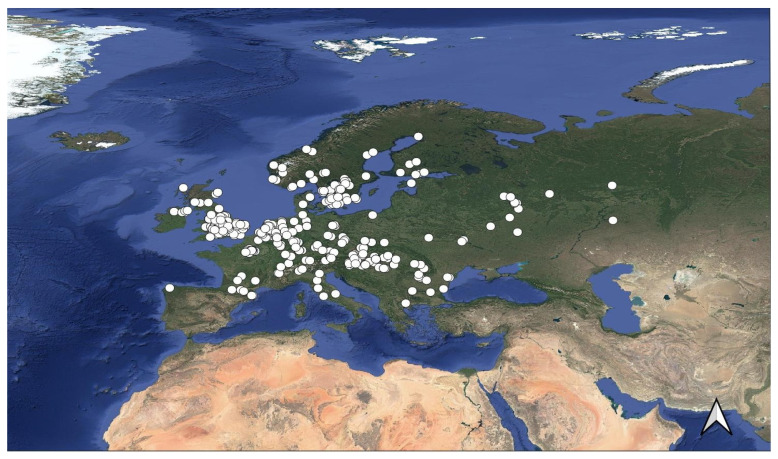
Distribution of *Limonia phragmitidis* used to create the Kernel Density Estimation for the bias file.

To account for bias in our presence data, we created a bias file using the distribution of *Limonia phragmitidis* (Schrank, 1781) (Figure 2). This species is widespread and common, making it a good proxy for sampling effort across the Western Palaearctic. The sampling bias file was constructed as follows: occurrence records for *L. phragmitidis* were downloaded from GBIF [36]. Only records with geographic coordinates were included, selecting both human observations and preserved specimen points. The dataset was then thinned using the R package rgbif [24], following the same procedure applied to *Dicranomyia* species. The final dataset consisted of 452 occurrence points. The bias file was generated in QGIS [28] using the Heatmap (Kernel Density Estimation) function, with a radius of 35 degrees and a quartic decay function. The resulting TIFF file was resampled to match the cell size of the environmental data, using the average resampling function.

## 3. Results

### 3.1. Evaluation of the Models

The best-performing models used 500,000 background points instead of the default 10,000 (Table 2). For *D. affinis*, the highest Test AUC (0.894) was obtained with the model using QP features and a regularization multiplier of 3. The lowest AICc (−63.171220) was achieved with the model built using L features and a regularization multiplier of 2. For *D. chorea*, the highest Test AUC (0.950) was produced by the model using QP features with a regularization multiplier of 1. The lowest AICc (−64.411760) was obtained with the model built with QP features and a regularization multiplier of 5. In the case of *D. mitis*, the model with the highest Test AUC (0.825) used L features with a regularization multiplier of 2. The lowest AICc (−124.497500) was obtained with the model built with LP features and a regularization multiplier of 3.

Differences in AICc values were remarkably high between models using the default number of background points and the models using 500,000 background points. The latter were much lower compared to the former. (Table 2).

Differences among Test AUC values were not so pronounced between the two groups of models, although slightly higher in favor of the models built using 500,000 background points (Table 2).

### 3.2. Dicranomyia affinis Models

**Figure 3:**
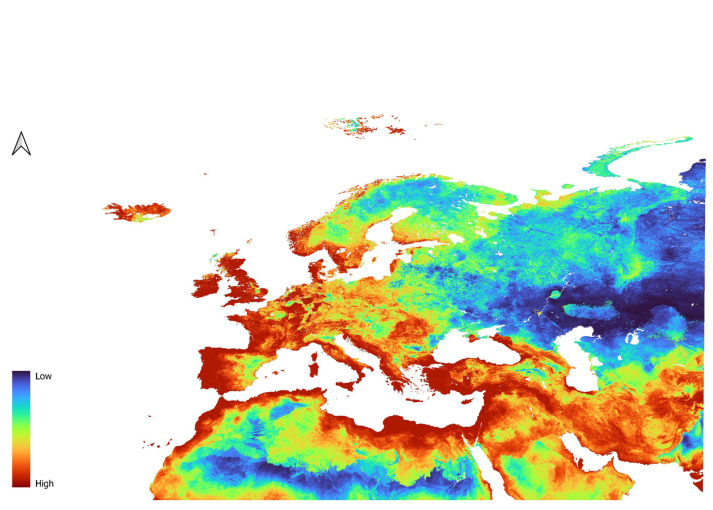
Prediction of suitability for *D. affinis* generated using the L feature and the Regularization parameter of 2.

**Figure 4:**
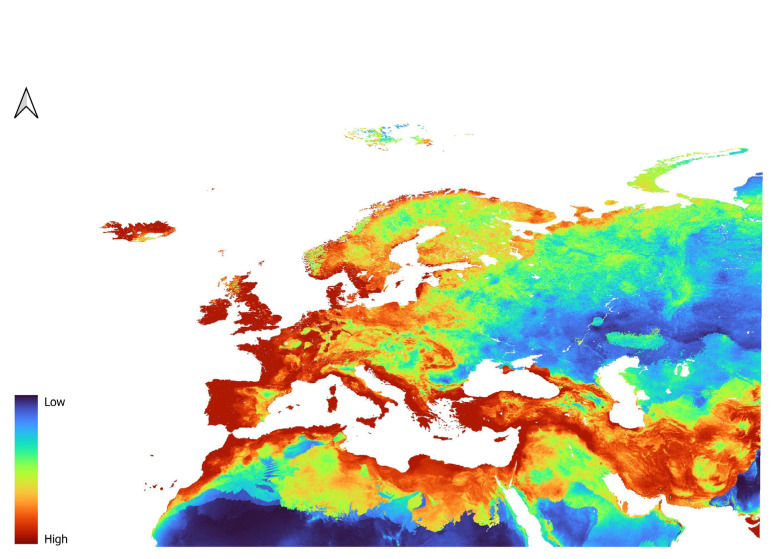
Prediction of suitability for *D. affinis* generated using QP features and the Regularization parameter of 3.

For *D. affinis*, the L2 model indicated high suitability in Western Europe (Figure 3), the Northern Arabian Peninsula, and the coastal regions of Northern Africa. In Western Europe, low to moderate suitability was observed in southeastern Spain and the Po Plain in Italy. In the Arabian Peninsula, low to moderate suitability was indicated within the central western and the southern central regions. Central Europe was generally classified as moderately to highly suitable, with most of Poland, Germany, and the western Baltic republics showing areas of high suitability. Greece, Turkey, and the Balkan countries were also identified as highly suitable. In Scandinavia, the model predicted high suitability along the coast, decreasing in inland and northern areas. Eastern Europe displayed low to moderate suitability, especially around the Russian Federation, while the Central Asian republics were considered largely unsuitable. The QP3 model (Figure 4) produced similar results, with some notable differences. Lower suitability was observed in the southern Arabian Peninsula and in inland areas of Northern Africa. Additionally, high-suitability areas were more widespread in France, southwestern Spain, and the U.K.

### 3.3. Dicranomyia chorea Models

**Figure 5:**
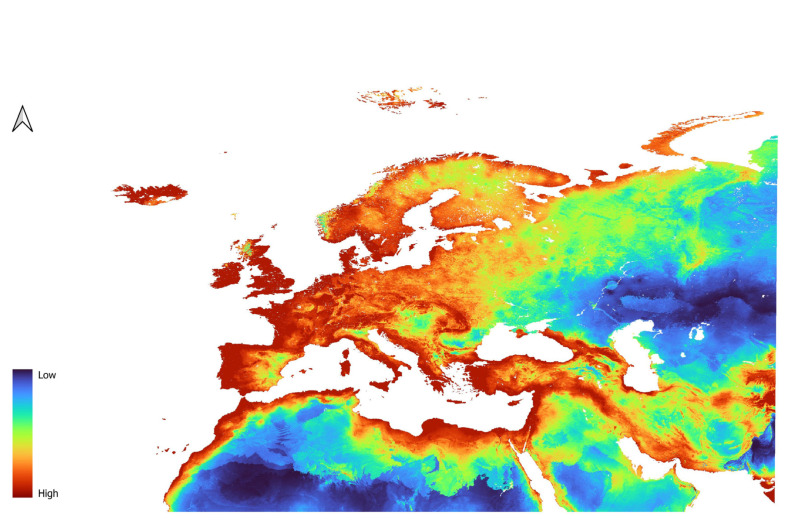
Prediction of suitability for *D. chorea* generated using QP features in combination with the regularization parameter of 5.

The models for *D. chorea* closely resembled those for *D. affinis*. The QP5 model (Figure 5) indicated very high suitability in most of Western Europe. In Northern Africa and the Arabian Peninsula, *D. chorea* showed high suitability only along the coast. High suitability was also observed in the western areas of the Baltic republics, gradually decreasing further east. This trend extended into the Russian Federation. Compared with *D. affinis*, suitability was more extended in the far eastern Russian Federation.

In contrast, the Central Asian republics were largely unsuitable for *D. chorea*. The Balkan countries, Greece, and Turkey were identified as highly suitable. Meanwhile, the Eastern Caucasian territories displayed scattered areas of moderate to low suitability.

**Figure 6:**
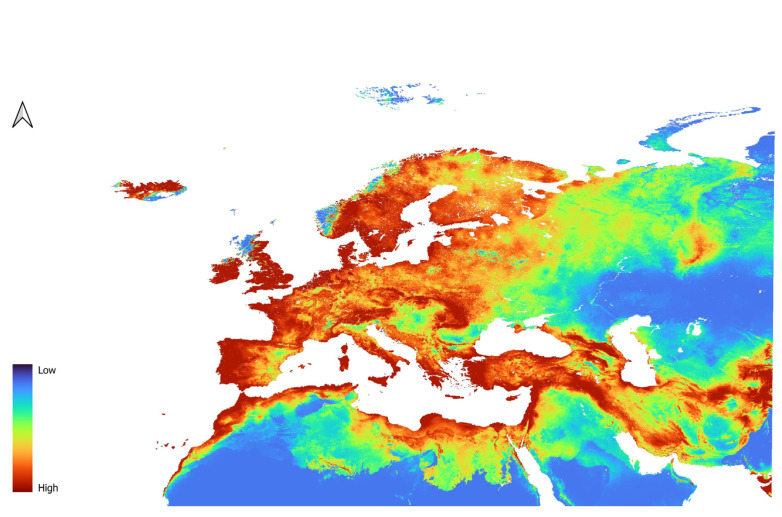
Prediction of suitability for *D. chorea* generated using the QP features in combination with the regularization parameter of 1.

The QP5 model for *D. chorea* was very similar to the QP1 model (Figure 6).

### 3.4. Dicranomyia mitis Models

**Figure 7:**
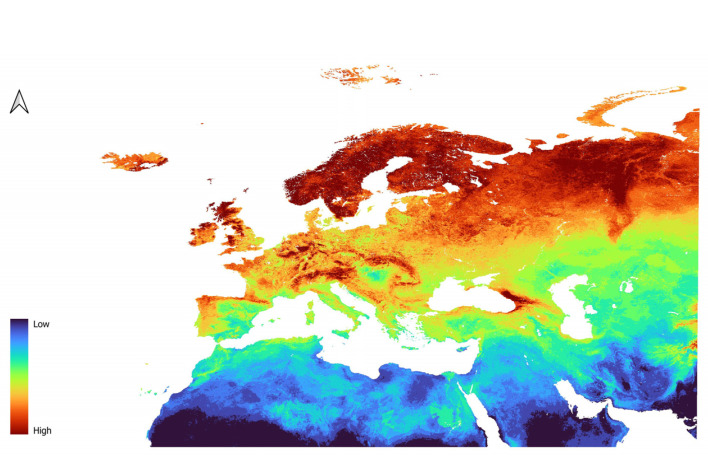
Prediction of suitability for *D. mitis* generated using the LP features in combination with the regularization parameter of 3.

**Figure 8:**
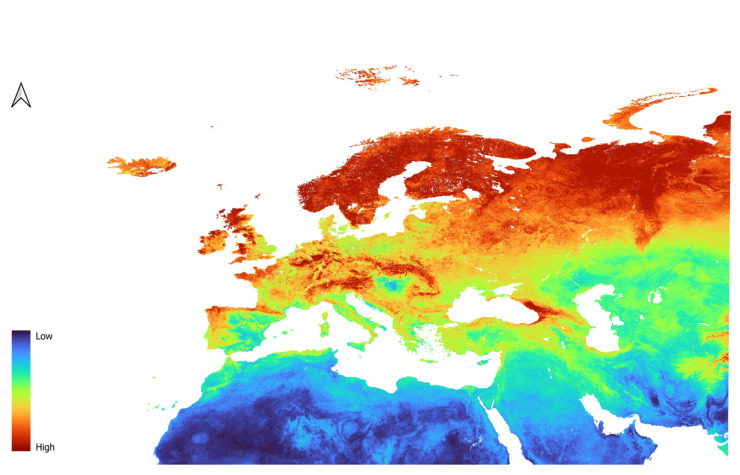
Prediction of suitability for *D. mitis* generated using the L features in combination with the regularization parameter of 2.

Models of *D. mitis* were different from the models of the previous species. The two models, LP3 (Figure 7) and L2 (Figure 8), provided very similar predictions. In fact, *D. mitis* distribution is predicted to be favored in Eastern Europe, reaching the far East, and much less so around the Mediterranean area and Western Europe in general.

Northern Africa and the Arabic Peninsula have low suitability for the species, as does most of the Caucasus. Concerning North Africa, slightly suitable conditions are only given in parts of the Atlas Mountain range.

South East Spain is also unsuitable, while Portugal and Northern Spain are moderately to highly suitable. Central Europe is predicted to have moderate to high suitability.

Italy, including the islands, the coast of Greece, and the Balkan republics are predicted to have low to moderate suitability.

### 3.5. Variable Importance

The most important contribution to the models were made by climatic variables for *D. affinis* and *D. chorea* while the most relevant variable in prediction of suitability for *D. mitis* was made by the variable pH (Table 3).

**Figure 9:**
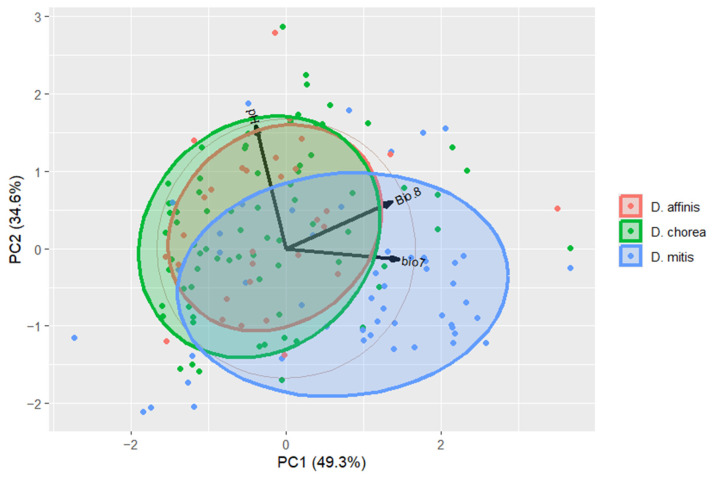
Principal Component analysis of the most influential variables (Bio7, Bio8, pH), showing overlap of *D. affinis* and *D. chorea* and partial overlap of *D. mitis* with the other species.

The PCA graph shows a complete overlap between the ellipses of *D. affinis* and *D. chorea*, while the ellipses of *D. mitis* show only a partial overlap between the latter and the former species (Figure 9). This indicates that the conditions of the most influential variables are very similar for *D. affinis* and *D. chorea*, but only partially so for *D. mitis* and the other two species.

## 4. Discussion

The primary objectives of this study were to explore the prediction of habitat suitability in less-sampled areas for three species of *Dicranomyia* crane flies and to assess possible differences in environmental preferences between the species.

Several unresolved issues persist in ENM, with no general consensus reached. Despite its widespread use, many uncertainties are still underappreciated [37].

One of the most critical concerns is the transferability of models. Given the current biodiversity crisis [38], the ability to predict species distributions across different geographic areas [6] or time periods [39,40] is essential for conservation and management. However, studies indicate that model transferability remains subject to considerable uncertainties [41,42].

A study by Qiao et al. [43] found that Maxent tends to overestimate the fundamental niche of species, leading it to predict larger suitable areas. Given that our occurrence points most likely represent an incomplete approximation of the fundamental niche, and considering the exploratory nature of this study, we opted for a liberal algorithm such as Maxent.

Data quality is another key source of uncertainty. A fundamental assumption is that species occurrences are proportional to their actual distribution [44]. However, this assumption is rarely met, as sampling efforts and published records exhibit significant biases [45,46]. Data collection often favors easily accessible areas, or knowledge about distribution is restricted to areas where most research takes place.

Although spatial filtering has been shown to reduce errors in Maxent models [35], our dataset contained insufficient occurrence points to apply this method. Instead, we accounted for bias by using the distribution of *L. phragmitidis*, a highly common species that provides a reasonable approximation of the geographic bias inherent in faunistic studies of Limoniidae.

Another crucial issue in SDM is model performance evaluation [47]. Ideally, independent test data should be used; however, test data should not share the same spatial bias as training data. This condition is not met when test data are withheld from the same sample as training data [48].

The most commonly applied metric for model evaluation is the AUC [49]. AUC is a discriminatory metric that assesses the model’s ability to predict specificity (proportion of correctly predicted absences) and sensitivity (proportion of correctly predicted presences) using a confusion matrix. However, AUC has been criticized for potentially yielding high values even for poorly fitted models [50] and for being influenced by the study area size, where larger areas tend to produce higher AUC values [51]. Warren and Seifert [14] compared various evaluation statistics and concluded that AICc was the most effective for small datasets (100 occurrence points vs. 1000 points).

Konowalik and Nosol [52] generated models using various datasets, ranging from narrow to broad occurrence ranges, in combination with different modeling tools, including Maxent, and multiple evaluation metrics. Their results indicated that diverse datasets could be useful for geographic prediction and that AUC performed well. Given the lack of a consensus on evaluation metrics and the automatic AUC output in Maxent, we used Test AUC in conjunction with AICc to assess our models.

Maxent is a presence-only algorithm, meaning it requires a comparison between environmental conditions at presence points and those in areas where the species is not recorded. As true absence data are unavailable, Maxent generates background points randomly across the study area. The default number of background points is set to 10,000. The number and distribution of background points significantly influence model predictions [53]. Increasing the number of background points improved model performance in our investigation. Since much of our study area was undersampled, increasing background points led to a better estimate of the environmental conditions across the study area.

Recently, ensemble modeling has been proposed as a tool to reduce variability in model outputs [54]. This approach consists of combining predictions from multiple modeling algorithms. The need to reduce variability among individual models, especially when predicting species distributions under climate change scenarios, has led researchers to investigate this method [55]. Such variability can, in fact, hinder the development of effective policy and management decisions.

Ensemble models have been employed to predict the distribution of medically important vectors [56] and are often advocated as a means of improving the accuracy of SDM [57]. However, evidence regarding their superiority over individual models remains mixed. For example, Montoya-Jiménez et al. [58] modeled the distributions of 17 pine species, coniferous taxa of high economic importance for timber production in Mexico, using nine SDM algorithms alongside an ensemble model. Although the ensemble model achieved the highest evaluation scores, the Maxent algorithm also performed strongly, and the predicted distributions were largely comparable between the two approaches.

In line with these findings, Zhu et al. [59] reported that ensemble models did not consistently outperform individual algorithms, highlighting their tendency to reflect the predictive capacity of the constituent models.

Interestingly, our study found differences in environmental preferences among the species studied.

The PCA of the most influential variables shows indeed only a partial overlap between *D. affinis*, *D. chorea*, and *D. mitis*, meaning that, based on our data, at least in part of the environmental space, the species differ.

Starý and Stubbs [13] briefly discussed the ecological preferences of *D. affinis* and *D. mitis*, largely based on field observations. *D. affinis* appears to favor acidic soils, whereas *D. mitis* prefers well-drained, slightly basic soils.

Differences in ecological preferences can fuel divergent selection and can result in speciation [60]. Nosil et al. [61] demonstrated that speciation could be driven by adaptation to different host plants in a species of walking-stick insect. Given the morphological similarity in *D. affinis* and *D. mitis* and based on the environmental differences seen in our results, it could be interesting to further investigate if the two species diverged recently and if their speciation is due to different ecological preferences. Testing this hypothesis would require experimental evidence from mate choice trials and an analysis of the DNA of both species. At the moment, this hypothesis is highly speculative but nonetheless intriguing.

Another interesting result of our study is that *D. affinis* and *D. chorea* have very low suitability scores around the Central Asian republics, while *D. mitis* has somewhat higher suitability in those areas.

Indeed, a faunistic report of the subfamily Limoniinae of East Kazakhstan did not report the three species that we modeled [62]. Kazakhstan is an arid country covered by large steppe areas with a temperate cold climate, which may require specific adaptations to these conditions. On the other hand, countries like Turkmenistan and Azerbaijan are warmer and mountainous, and indeed, suitability scores are higher for the three species studied. *Dicranomyia mitis* is the only species predicted to have low to moderate suitability in Central Asia, meaning that it could be the species better adapted of the three studied to the drier and colder winters of those areas.

Collecting additional data on distributions of the species in areas where only *D. mitis* is predicted to have suitable conditions, or where the species have less suitable conditions, could confirm or refute the differences seen in the models.

## 5. Conclusions

The results of this study indicate that *D. affinis* and *D. chorea* share similar habitat preferences, favoring warmer environments, in contrast to *D. mitis*. Consequently, *D. affinis* and *D. chorea* are likely to find suitable conditions in Western Europe, whereas *D. mitis* is predicted to thrive in Eastern Europe, Scandinavia, and, to a lesser extent, the Mediterranean. Notably, the closely related species *D. affinis* and *D. mitis* appear to differ in at least part of the environmental space examined. Additional experimental and genetic studies, along with expanded sampling, particularly in the Eastern Palaearctic, are needed to test this hypothesis. Overall, the findings seem to support the recognition of *D. affinis* and *D. mitis* as distinct taxonomic entities. However, given the uncertainties inherent to SDM, these results should be interpreted with caution.

## Figures and Tables

**Table 1:** Environmental variables used in this study.

Environmental Variables	Description	Source
Bio6	Min temperature of Coldest Month	WorldClim
Bio7	Temperature Annual Range	WorldClim
Bio8	Mean Temperature of Wettest Quarter	WorldClim
Bio13	Precipitation of Wettest Month	WorldClim
Bio15	Precipitation Seasonality (Coefficient of variation)	WorldClim
Clay	Proportion of clay particles (<0.002 mm) in the fine earth fraction (g/kg) at depth 15/30 cm	Soilgrids
Nitrogen	Total nitrogen (cg/kg) at depth 15/30 cm	Soilgrids
phh2o	Soil pH	Soilgrids
Sand	Proportion of sand particles (>0.05/0.063 mm) in the fine earth fraction (g/kg) at depth 15/30 cm	Soilgrids

**Table 2:** Results of AICc and Test AUC evaluation metrics. L = Linear, Q = quadratic, P = product Regularization parameters = 1,2,3,4,5. 10k = models with 10,000 background points; 500k= models with 500,000 background points. λ = number of parameters. In yellow best performing models.

D. mitis			D. chorea				D. affinis		
λ	AiCc	Test AUC	λ	AICc	Test AUC	λ	AICc	Test AUC	Model
7	244.6519	0.808	9	262.678500	0.910	7	100.092800	0.823	L1_10k
5	237.540500	0.824	8	264.809600	0.910	5	93.570330	0.836	L2_10k
4	236.915000	0.823	8	264.809600	0.910	5	93.758980	0.839	L3_10k
4	236.975900	0.820	8	265.871800	0.909	3	88.329820	0.832	L4_10k
4	237.043300	0.818	7	264.082200	0.907	3	88.466770	0.808	L5_10K
7	−114.563200	0.809	9	−54.147510	0.915	7	4.459933	0.823	L1_500k
6	−117.191100	0.825	8	−56.724220	0.916	5	−63.171220	0.835	L2_500k
4	−122.165000	0.823	8	−56.731830	0.915	5	0.489773	0.841	L3_500k
4	−122.153900	0.821	8	−56.760000	0.914	4	−2.496222	0.837	L4_500k
4	−122.143400	0.819	7	−59.322370	0.912	3	−5.193853	0.819	L5_500k
7	244.947100	0.791	9	266.614400	0.928	8	102.453500	0.849	Q1_10k
6	241.887700	0.810	8	265.435400	0.929	7	99.925430	0.838	Q2_10k
4	236.780600	0.819	8	266.387800	0.928	6	96.902220	0.831	Q3_10k
4	236.825000	0.816	8	267.009200	0.928	5	93.853360	0.848	Q4_10k
3	234.522700	0.814	8	267.450400	0.924	4	91.024330	0.852	Q5_10k
7	−114.513700	0.790	9	−54.279050	0.932	8	10.786890	0.853	Q1_500k
6	−117.167500	0.814	8	−56.874890	0.933	7	7.012010	0.841	Q2_500k
4	−122.152500	0.819	8	−56.950410	0.933	7	6.908883	0.835	Q3_500k
4	−122.146500	0.816	8	−57.035180	0.932	5	0.328777	0.847	Q4_500k
3	124.490600	0.814	8	−57.104580	0.929	4	−2.555656	0.854	Q5_500k
8	247.125600	0.820	15	290.498300	0.937	10	112.573800	0.884	LP1_10k
5	239.367900	0.818	10	271.645600	0.937	10	112.664000	0.873	LP2_10k
3	234.730600	0.812	7	264.929200	0.931	6	97.043540	0.877	LP3_10k
3	234.764400	0.811	5	260.878300	0.927	4	91.126160	0.869	LP4_10k
3	234.794500	0.811	5	261.476000	0.926	3	88.510480	0.863	LP5_10k
8	−111.790300	0.824	14	−36.334200	0.941	10	32.729570	0.888	LP1_500k
5	−119.728800	0.821	10	−48.845840	0.941	11	25.108980	0.879	LP2_500k
3	−124.497500	0.813	7	−59.258310	0.936	7	6.969385	0.883	LP3_500k
3	−124.492800	0.813	5	−64.248550	0.932	4	−2.524094	0.871	LP4_500k
3	−124.489200	0.813	5	−64.281020	0.931	3	−5.200798	0.866	LP5_500k
10	253.519000	0.797	10	268.262700	0.934	9	106.090700	0.847	LQ1_10k
8	247.210700	0.820	10	269.207500	0.930	7	99.732720	0.843	LQ2_10k
4	236.744800	0.822	10	270.577300	0.927	7	99.845550	0.864	LQ3_10k
4	236.792300	0.820	8	266.133800	0.926	6	96.583660	0.886	LQ4_10k
4	236.847900	0.817	8	266.648700	0.924	5	93.686940	0.883	LQ5_10k
9	−108.846600	0.796	12	−46.288390	0.939	9	14.700140	0.849	LQ1_500k
8	−111.733600	0.823	10	−51.678820	0.935	7	7.154227	0.844	LQ2_500k
4	−122.160200	0.822	9	−54.226530	0.932	7	6.962915	0.866	LQ3_500k
4	−122.154800	0.820	8	−56.937470	0.931	6	3.472355	0.889	LQ4_500k
4	−122.151500	0.817	8	−57.040290	0.929	5	0.324897	0.889	LQ5_500k
7	244.295700	0.819	13	275.294900	0.945	10	111.188700	0.882	QP1_10k
5	239.164300	0.818	11	273.452600	0.940	8	103.588400	0.881	QP2_10k
3	234.495800	0.818	8	267.044100	0.934	8	104.149200	0.887	QP3_10k
3	234.557300	0.819	6	262.877900	0.932	5	94.050960	0.849	QP4_10k
3	234.620900	0.819	5	261.166300	0.931	3	88.561700	0.833	QP5_10k
8	−111.762000	0.822	14	−38.408350	0.950	10	20.116190	0.885	QP1_500k
5	−119.711500	0.823	11	−48.739510	0.945	8	10.760050	0.885	QP2_500k
3	−124.481100	0.820	8	−57.059590	0.939	8	10.693920	0.894	QP3_500k
3	−124.479000	0.821	6	−62.042200	0.937	6	3.490096	0.863	QP4_500k
3	−124.477100	0.821	5	−64.411760	0.936	3	−5.232400	0.838	QP5_500k

**Table 3:** Variable importance from the Maxent output calculated by the Permutation importance test and the Jackknife test.

*D. affinis*	*D. chorea*	*D. mitis*
Bio7; Bio8	Bio7; Bio8	ph

## Data Availability

The data supporting the results are available from the corresponding author upon request.

## Supplementary Materials

The following supporting information can be downloaded at https://www.mdpi.com/article/10.3390/insects16101022/s1, Table S1: Georeferenced points from the literature.

## Abbreviations

The following abbreviations are used in this manuscript:

Akaike Information Criterion corrected for small sample size	AICc
Area Under the Curve of the Receiver Operating Characteristic	AUC
Ecological Niche Modeling	ENM
Global Biodiversity Information Facility	GBIF
Linear feature of Maxent	L
Quadratic feature of Maxent	Q
Linear Product features of Maxent	LP
Linear Quadratic features of Maxent	LQ
Principal Component Analysis	PCA
Quadratic Product features of Maxent	QP
Naturalis Biodiversity Center, Leiden, The Netherlands	RMNH
Spatial Distribution Modeling	SDM
10,000 background points	10k
500,000 background points	500k
