# Supplementary material for: Exploring Potential Distribution and Environmental Preferences of Three Species of Dicranomyia (Diptera: Limoniidae: Limoniinae) Across the Western Palaearctic Realm Using Maxent
Source: Insects. 2025 Oct 2;16(10):1022. doi: 10.3390/insects16101022 (PMC12565149; doi:10.3390/insects16101022)
Supplement: Supplementary file 1 [file insects-16-01022-s001.zip › insects-3851588-supplementary.pdf]

Table S1. Georeferenced points from the literature.

Species	Country	Location	Latitude	Longitude	Radius (m)	Source
D. affinis	France	Vouzeron, Foret de Vouzeron	47.267 N	2.198 E		[1]
D. affinis	France	Les Alleuds	47.315 N	-0.412 W	2600	[2]
D. affinis	Italy	Monti 3 km SE, road 389 (maquis) 7.v.2014	40.800 N	9.350 E		[3]
D. affinis	Italy	Mazzinaiu nr. Alà dei Sardi, 3.5 km NE , 7.v.201	40.733 N	9.400 E		[3]
D. affinis	Morocco	Jbel Bouhachem, Daya Mtahen, marsh, 27.iv.2012	35.184 N	-5.346 W	3500	[3]
D. affinis	Morocco	Jbel Khizana, Azghar, brook	35.188 N	-3.900 W	1700	[3]
D. affini	Morocco	Jbel Zem Zem, marsh, 25.iv.2014	35.701 N	-5.627 W	2300	[3]
D. affinis	Morocco	Oued Amsemlil (Site 1)	35.260 N	-5.433 W		[4]
D. affinis	Morocco	Daya Mtahen	35.270 N	-5.435 W		[4]
D. affinis	Morocco	Oued Tkaraâ	35.268 N	-5.430 W		[4]
D. affinis	Morocco	Marj El Kheyl	35.266 N	-5.437 W		[4]
D. affinis	Morocco	Aïn Sidi Brahim Ben Arrif (Site1)	35.340 N	-5.542 W		[4]
D. affinis	Morocco	Aïn Sidi Brahim Ben Arrif (Site2)	35.340 N	-5.542 W		[4]
D. affinis	Morocco	Daya Tazia	35.347 N	-5.552 W		[4]
D. affinis	Morocco	Aïn El Maounzil	35.076 N	-5.173 W		[4]
D. affinis	Morocco	Aïn El Malâab	35.092 N	-5.157 W		[4]
D. affinis	Morocco	Daya near Aïn Afersiw	35.101 N	-5.339 W		[4]
D. affinis	Morocco	Daya Mtahe	35.270 N	-5.436 W		[4]
D. affinis	Morocco	Aïn Bab Tariouente	35.019 N	-5.010 W		[4]
D. affinis	Netherlands	Elim Plangebied - Nieuwlande Dalerend (DR)	52.685 N	6.622 E		[5]
D. affinis	Russia	Beregoviye Syuresi, Mordovia, Russia	54.759 N	45.612 E		[6]
D. affinis	Spain	Castilla y Leon Candin	42.817 N	-6.726 W		[1]
D. affinis	Spain	Alto de La Farrapona	43.065 N	-6.111 W	3000	[7]
D. affinis	United Kingdom	Flintshire, Ffrith,	53.093 N	-3.070 W		[3]
D. affinis	United Kingdom	South Devon, Dartmouth	50.352 N	-3.590 W		[3]
D. affinis	United Kingdom	East Sussex, Frant	51.096 N	0.270 E		[3]
D. affinis	United Kingdom	Bedfordshire, Barton	51.962 N	-0.425 W		[3]
D. affinis	United Kingdom	Bedlock	51.987 N	-0.188 W		[3]
D. affinis	United Kingdom	Leigh Woods	51.463 N	-2.640 W	500	[8]
D. chorea	Azerbaijan	Gədəbəy district, Gədəbəy, bushy brook and seep S of village	40.549 N	45.718 E		[9]
D. chorea	Azerbaijan	Şəki district, Kiş, karst spring and brook by Galarsan ruin	41.265 N	47.293 E		[9]
D. chorea	Czech Republic	Jizerka	50.818 N	15.345 E	1500	[10]
D. chorea	Czech Republic	Meandry Smědé	50.990 N	15.031 E	1300	[10]
D. chorea	France	Marans, Charent-Maritime	46.340 N	-1.038 W	3000	RMNH
D. chorea	Georgia	Kakheti region, Napareuli, Lopota Lake and its inlet brook	42.057 N	45.527 E		[9]

D. chorea	Georgia	Mtskheta-Mtianeti region, Meneso, Aragvi River	42.229 N	44.673 E		[9]
D. chorea	Israel	Tel Dan	33.248 N	35.651 E	1000	[11]
D. chorea	Israel	Nussbaum, Horeshat Tal	33.221 N	35.630 E	1000	[11]
D. chorea	Israel	Panyas [Banias, Banyas]	33.242 N	35.682 E	1000	[11]
D. chorea	Israel	Bet Hillel	33.212 N	35.602 E	2400	[11]
D. chorea	Israel	Panyas	33.247 N	35.694 E		[12]
D. chorea	Israel	Har Meron Reserve, ʼEn haZaqen	32.967 N	35.412 E		[12]
D. chorea	Italy	Bivere di Cesaro	37.955 N	14.714 E	2500	RMNH
D. chorea	Italy	Belvi	39.962 N	9.184 E	1000	[13]
D. chorea	Italy	Aspromonte, Cascate tre limiti	38.139 N	15.860 E		Collected on 06/06/2024
D. chorea	Malta	Baħrija	35.894 N	14.350 E	2000	[14]
D. chorea	Malta	Buskett	35.860 N	14.398 E	2000	[14]
D. chorea	Morocco	Oued Sidi Fares	31.234 N	-7.880 W		[4]
D. chorea	Morocco	Oued Farda	35.233 N	-5.173 W		[4]
D. chorea	Morocco	Oued Kelâa	35.238 N	-5.174 W		[4]
D. chorea	Morocco	Aïn Ras El Ma,	35.114 N	-5.190 W		[4]
D. chorea	Morocco	Oued Jnane Niche	35.284 N	-4.854 W		[4]
D. chorea	Morocco	Cascade Chrafate	35.067 N	-5.107 W		[4]
D. chorea	Morocco	Maison forestière	35.135 N	-5.138 W		[4]
D. chorea	Morocco	Oued El Kanar	35.287 N	-4.993 W		[4]
D. chorea	Morocco	Tributary of Oued Zarka	35.520 N	-5.341 W		[4]
D. chorea	Netherlands	Etten	51.910 N	6.331 E	3500	RMNH
D. chorea	Poland	Wyżyna Krakowsko-Wieluńska	50.207 N	19.829 E	1500	[13]
D.chorea	Portugal	Algarve: Vila do Bispo, 4 km NE, Lagoa Funda, marsh (126 m)	37.117 N	-8.900 W		[15]
D. chorea	Portugal	Serra de Monchique, Foia Mt, springs and marshes	37.312 N	-8.600 W		[15]
D. chorea	Portugal	Camara de Lobos, Jardim de Serra	32.689 N	-16.990 W	1600	RMNH
D. chorea	Russia	Bashkortostan Respublika;Beloretsk, Mata River	54.004 N	58.464 E		[1]
D.chorea	Spain	Mallorca: Fangar nr. Campanet, Torrent Massana (nr. brook)	39.808 N	2.977 E		[15]
D. chorea	Spain	Binibona nr Selva (nr brook)	39.784 N	2.944 E		[15]
D. chorea	Spain	Aguaecebos de Gil Cobo trail	38.976 N	-2.894 W		[16]
D. chorea	Switzerland	Gandria	46.006 N	9.002 E	2700	[13]
D. mitis	Austria	Niederösterreic: Gutenstein env., 17.v.1994	47.876 N	15.890 E	795	[3]
D. mitis	Bulgaria	Sofia, Vitosha Mt., 8.v.1981	42.550 N	23.250 E	1600	[3]
D. mitis	Bulgaria	Ostar Kamak, 3.v.1980	41.888 N	25.850 E	628	[3]
D. mitis	Czech Republic	rubý Jeseník Mts, Petrovy kameny, peat-bog	50.067 N	17.233 E	2500	[3]
D. mitis	Czech Republic	Zvíčina [hill] nr. Dvůr Králové, 24.v.1972,	50.454 N	15.697 E	2120	[3]
D. mitis	Czech Republic	Bukovec nr. Jablunkov, 25.vi.1997	48.713 N	21.146 E	2800	[3]
D. mitis	Czech Republic	Pustevny (1000 m), 26.vi.1985	49.490 N	18.266 E	2500	[3]

D. mitis	Czech Republic	Lešná nr. Zlín (= nr. Gottwaldov), 23.v.1967	49.518 N	17.931 E	2500	[3]
D. mitis	France	Fources, observation by Micha d' Oliveira	43.993 N	0.208 E	5	[17]
D. mitis	France	La Jaille-Yvon	47.730 N	-0.689 W	3600	[18]
D. mitis	France	Montreuil Juigné	47.542 N	-0.619 W	3000	[18]
D. mitis	Italy	Trafoi	46.551 N	10.508 E	1000	[19]
D. mitis	Italy	Val Ferret	45.845 N	7.014 E	2000	[20]
D. mitis	Netherlands	Geuldal - Zuid van Epen, observation by Micha d' Oliveira	50.764 N	5.928 E	10	[21]
D. mitis	Netherlands	Yerseke, observation by Niels Jan Dek	51.499 N	4.041 E	10	[17]
D. mitis	Slovakia	Poľana Mts, Čierny Potok (700 m), 25.v.2005	48.299 N	19.938 E	2300	[3]
D. mitis	Slovakia	Ruský Potok, 14.vi.1991	49.029 N	22.411 E	2950	[3]
D.mitis	Slovakia	Malá Fatra Mts, Biela (700 m), 28.v.1992	49.241 N	19.136 E	1400	[3]
D. mitis	Spain	La Colniella	43.443 N	-6.321 W	1000	[7]

References:

1. Kolcsár, L.-P.; Oosterbroek, P.; Gavryushin, D.; Olsen, K. M.; Paramonov, N.; Pilipenko, V.; Starý, J.; Polevoi, A.; Lantsov, V.; Eiroa, E.; et al. Contribution to the Knowledge of Limoniidae (Diptera: Tipuloidea): First Records of 244 Species from Various European Countries. *Biodivers. Data J.* **2021**, *9*, e67085. https://doi.org/10.3897/BDJ.9.e67085.
2. Quindroit, C. Liste des Tipuloidea des Pays de la Loire : rectifications et ajouts (Diptera). *L' Entomologiste* **2022,** *78*, 13–28.
3. Starỳ, J.; Stubbs, A. E. Five Species under *Dicranomyia (Dicranomyia) mitis* (Meigen, 1830)(Diptera, Limoniidae). *Zootaxa* **2015**, *3964*,321–334. https://doi.org/10.11646/zootaxa.3964.3.2.
4. Driauach, O.; Belqat, B. Additions to the Limoniidae and Pediciidae Fauna of Morocco, with an Updated Checklist (Diptera, Tipuloidea). *ZK* **2016**, *563*, 129–146. https://doi.org/10.3897/zookeys.563.7384.
5. Dek, N.-J. De steltmug *Dicranomyia affinis* nieuw voor Nederland (Diptera: Limoniidae). *Nederlandse Faunistische Mededelingen* **2021**, No. 57, 71–74.
6. Oboňa, J.; Esin, M.; Barták, M.; Dvořák, L.; Ježek, J.; Ruchin, A. New and Interesting Records of Flies (Diptera) in the Regions of European Russia. *Far East. Entomol.* **2024**, *7* (510).
7. Keresztes, L.; Martinez, J. Observaciones sobre los Limoniidae y Pediciidae de Asturias (norte península ibérica) (Diptera, Tipuloidea). *Boletin Asoc. Espanola Entomol.* **2022**, 46, 191–198.
8. Kramer, J. In the Footsteps of Henri Audcent. *Bulletin of the Dipterists Forum 76; Cranefly News. Dipterists Forum Cranefly Recording Scheme Newsletter*. 2013, pp 5–8.
9. Starỳ, J.; Oboňa, J. Further Records of Pediciidae and Limoniidae (Diptera) from Azerbaijan and Georgia. *Pol. J. Entomol.* **2020**, *89* (3), 124–141.
10. Starý, J.; Vonička, P. Limoniidae and Pediciidae (Diptera: Tipulomorpha) of the Jizerské Hory Mts, Frỳdlant Region, and Liberec Environs (Northern Bohemia, Czech Republic). *Muzea Prir. Vedy* **2018**, 36, 45–83.
11. Starỳ, J.; Freidberg, A. The Limoniidae of Israel (Diptera). *Isr. J. Entomol.* **2007**, *37*, 301–357.
12. Starý, J. Additions to the Limoniidae of Israel (Diptera). *Isr. J. Entomol.* **2014a**, 43, 149–157.
13. Starý, J.; The identity of *Dicranomyia (Dicranomyia) luteipennis* Goetghebuer (Diptera,Limoniidae). *Zootaxa.* **2009,** 2155(1). https://doi.org/10.11646/zootaxa.2155.1.5
14. Ebejer, M. The Craneflies (Diptera, Tipulidae and Limoniidae) and Winter Gnats (Diptera, Trichoceridae) of Malta. *Bull. Entomol. Soc. Malta* **2015**, No. 7, 51–55.
15. Starỳ, J. Some Records of Limoniidae and Pediciidae (Diptera) from Portugal and Spain. *Acta Musei Silesiae Sci. Nat.* **2014b**, *63* (1), 83–95.
16. Hancock EG, Hewitt ES (2020). Craneflies (Diptera, Tipuloidea) trapped by the leaves of long-leaved Spanish butterwort, *Pinguicula vallisneriifolia* Webb (Lentibulariaceae). Diptera Digest 27: 45–49

17. Observations.org available at https://observations.org/ (accessed on 09/12/2024)
18. Quindroit, C. Une première liste des Tipuloidea des Pays de la Loire et addition d'une espèce de Limoniidae à la faune de France (Diptera). *L'Entomologiste* **2020**, 76, 5–48.
19. Podenas, S.; Podeniene, V. Tipuloidea Excl. Tipulidae. *Ziegler J Ed Diptera Stelviana Stud. Dipterol.* **2008**, *1* (Supplement 16), 345–354.
20. Starỳ, J.; Oosterbroek, P. Review of the Limoniidae and Pediciidae of Italy. *Fragm. Entomol. Roma* **1996**, 28, 51–95.
21. Waarneming.nl available at https://waarneming.nl/ (accessed on 07/12/2024)

Abbreviations

RMNH= Naturalis Biodiversity Center, Leiden, The Netherlands
